# Preliminary Study on Disinfectant Susceptibility/Resistance Profiles of Bacteria Isolated from Slaughtered Village Free-Range Chickens in Nairobi, Kenya

**DOI:** 10.1155/2021/8877675

**Published:** 2021-02-20

**Authors:** Igizeneza Acsa, Bebora Lilly Caroline, Nyaga Philip Njeru, Njagi Lucy Wanjiru

**Affiliations:** College of Agriculture and Veterinary Sciences, Faculty of Veterinary Medicine, Department of Veterinary Pathology,Microbiology and Parasitology, University of Nairobi, Nairobi, Kenya

## Abstract

Disinfectants are regularly used for cleansing poultry slaughterhouses to control microorganisms. However, the microorganisms such as bacteria are developing resistance to disinfectant(s) and complicate control of bacterial infections. The aim of this study was, therefore, to determine disinfectant susceptibility/resistance patterns manifested by bacteria (to commonly used disinfectants), which were isolated from intestines of slaughtered indigenous chickens in Nairobi, Kenya. The method used was agar well diffusion, and the six disinfectants (their active ingredients are in brackets) tested were as follows: Kupacide^®^ (glutaraldehyde; benzalkonium chloride); TH4+^®^ (didecyl dimethyl ammonium HCl; dioctyl dimethyl ammonium HCl; octyl decyldimethyl ammonium HCl; alkyl dimethyl ammonium HCl; and glutaraldehyde); Noro cleanse^®^ (glutaraldehyde; coco-benzyl-dimethyl-ammonium chloride); Dettol^®^ (chloroxylenol); Savlon^®^ (chlorhexidine gluconate; cetrimide; and N-propylalcohol); and Jik^®^ (sodium hypochlorite). At recommended user concentration by the manufacturer, isolates showed various resistance to the respective disinfectants. *E. coli* isolates were resistant to five of the tested disinfectants (Jik^®^, TH4+^®^, Noro cleanse^®^, Dettol^®^, and Kupacide^®^); however, they were susceptible to Savlon^®^; *Staphylococcus* isolates were resistant to disinfectants to Jik^®^ and TH4+^®^ and susceptible to the rest disinfectants; *Streptococcus* isolates were only resistant to Jik^®^ and susceptible to the remaining disinfectants. Some *E. coli* and *Staphylococcus* isolates showed resistance to more than one disinfectant. This study has demonstrated resistance of the bacterial isolates to various disinfectants at recommended user concentrations, although some of them were susceptible at higher concentration(s) and lower concentrations. This will interfere with the cleansing of the respective premises, resulting in contaminated products, which may end-up causing disease in the humans consuming them. Hence, it is recommended that one ascertains the efficacy of respective disinfectant by carrying out disinfectant susceptibility testing to know the effective ones and the appropriate concentration to use.

## 1. Introduction

Disinfectants are chemical agents which are used for decontamination of surfaces and other inanimate objects applied in different fields, including in poultry production [1]. They are used to kill pathogenic microorganisms or reduce them to acceptable levels; some can also destroy their spores [1]. Disinfection does not necessarily kill all microorganisms but reduces them to a level acceptable for a defined purpose [2, 3]. Potentially toxic products can be applied to inanimate objects or surfaces, whereas for disinfection of human tissues, only the less toxic disinfectants (antiseptics) can be considered [3]. The following are a few examples of disinfectants: phenolic compounds, alcohols, chlorhexidine, chlorine compounds, formaldehyde, glutaraldehyde, hydrogen peroxide, iodophore, peracetic acid, and quaternary ammonium compounds.

Disinfectants are used for biosecurity and biosafety purposes; they help in controlling disease-causing pathogens [4]. They are used extensively in human activities for cleansing purposes and in intensive poultry farms as part of hygienic practices, for prevention of diseases [5]. The use of disinfectants for sanitation in food industries is very important because it ensures that there are no viable cells which can grow and multiply and contaminate the food materials [4, 6]. However, this practice must be done prudently because the chemicals used as disinfectants can also cause harm to humans.

So, the selection of disinfectants to be used must depend on their efficacy, safety, and rinsability. Use of disinfectants helps in reducing the surface microorganisms, hence reducing the chance of spreading foodborne illness [3]. Disinfectants reduce microbial loads by working on different target sites resulting in membrane disruption, metabolic inhibition, and lysis of the particular cell [5, 7].

Bacterial resistance to disinfectants depends on intrinsic factors and other environmental conditions [6]. It is, therefore, important to conduct the disinfectant susceptibility test in order to select the effective ones; noting that, in most cases, the more active a disinfectant is, the more toxic it is [3]. Resistance to disinfectants can occur and can be towards a single disinfectant or to several disinfectants [8, 9].

There are several methods used to test for disinfectant effectiveness, but the one mostly used is diffusion technique, where wells are dug into the inoculated agar and are filled with the respective disinfectant [2, 10]. In this investigation, different disinfectants were tested against bacteria isolated from intestines of slaughtered indigenous chickens using the agar well diffusion method.

## 2. Materials and Methods

### 2.1. Study Design, Sample Collection, and Processing

This was a cross-sectional investigation, where intestines obtained from indigenous chickens brought for slaughter were collected from three different slaughterhouses (Kariokor, Burma, and Kangemi) in Nairobi, Kenya. The total number of samples collected was one hundred and twenty (120); 40 were taken from each slaughterhouse.

The intestines were collected aseptically, put in separate sterile universal bottles, and transported in a cool box to the bacteriology laboratory, Department of Veterinary Pathology, Microbiology and Parasitology, University of Nairobi, for processing. At the laboratory, standard bacteriological methods were used for bacterial isolation and characterization [11]. For each of the three isolated bacterial genera, *Staphylococcus*, *Streptococcus*, and *Escherichia*, five isolates from each slaughterhouse were tested for their disinfectant susceptibility/resistance patterns, with respect to six disinfectants which are commonly used in poultry intensive production units/farms, hospitals, laboratories, and for hand washing using agar well diffusion technique [2]. Reference strains included ATCC 25923 *Staphylococcus aureus* and ATCC 25922 *Escherichia coli*.

### 2.2. Disinfectant Susceptibility/Resistance Testing and Data Analysis

Six disinfectants Kupacide^®^; TH4+^®^; Noro cleanse^®^; Dettol^®^; Savlon^®^; and Jik^®^, which are commonly used in poultry intensive production units/farms, hospitals, and laboratories and for hand washing, were used.  Table 1 shows detailed information on the tested disinfectants, including their active ingredients.

Tested disinfectants were recommended by the manufactures to have different antimicrobial effects. Dettol^®^ has effect against bacteria such as *E. coli. Staphylococcus aureus*, *Enterococcus hirae*, *Salmonella*, and *Vibrio cholera*; fungus such as C*andida albicans*; and viruses such as influenza A, herpes simplex, SARS, hepatitis C, and avian H1N1/H5N1. Kupacide^®^ is effective in killing bacteria, mycoplasma, fungi, coccidian, and inactivating viruses. Savlon^®^ is a professional germ killer that kills over 99.9% of germs. TH4+^®^ is known to have a broad spectrum activity against bacteria, viruses, and fungi; its antibacterial activity is against *Escherichia coli*, *Bordetella bronchiseptica*, *Campylobacter jejuni*, *Enterococcus faecium*, *Listeria monocytogenes*, *Erwinia* spp., *Leptospira interrogans*, *Mycobacterium*, and *Mycoplasma hyopneumoniae*. According to the manufacturer of Noro cleanse^®^, the disinfectant is effective against bacteria, virus, and fungi. Jik^®^ as a disinfectant is used to sanitize and kill gems.

Disinfectant susceptibility testing was done using the agar well method, as described by Turkun et al. [12] and Njagi et al. [2] with little modification. Muller-Hinton plates (Oxoid, Basingstoke, United Kingdom), with the same agar depth (6 mm), were seeded with bacterial suspensions whose turbidity was adjusted to match that of 0.5 MacFarland nephelometer tube. Wells were then dug using sterile 6 mm diameter well puncher and filled with respective disinfectants at different concentrations, and incubated up-side-up overnight at 37°C prior to reading. The inhibition diameters were interpreted according to Njagi et al. [2] since there are no established cutoff points for the specific disinfectants. Diameter measurements below or equal to 10 mm were considered as resistant (R); those beyond 10 mm were considered susceptible (S).

Each disinfectant was diluted according to the manufacturer's recommended concentration, given as concentration 3^∗^ in Table 2 and other dilutions above x2 and x4 and below x1/2 and x1/4 the recommended user concentration, using sterile normal saline. The results were analysed using Statistical Package for the Social Sciences (SPSS); chi-square was used to test the association of disinfectant resistant isolates with their respective slaughter houses at a *p* value of 0. 05.

The recommended concentration by the manufacturer was as follows: for Kupacide^®^, Th4+^®^, and Noro cleanse^®^, the recommended concentration was 0.25% for each; for Dettol^®^, Savlon^®^, and Jik^®^, it was 5%, 6%, and 2.27%, respectively. Other concentrations were also tested with respective to recommended concentration. 1 (x1/4) was the lowest concentration; 2 (x1/2) was the next lower concentration; 4 (x2) was twice the concentration recommended by the manufacture, and 5 (x4) was the highest concentration.

## 3. Results

**Figure 1:**
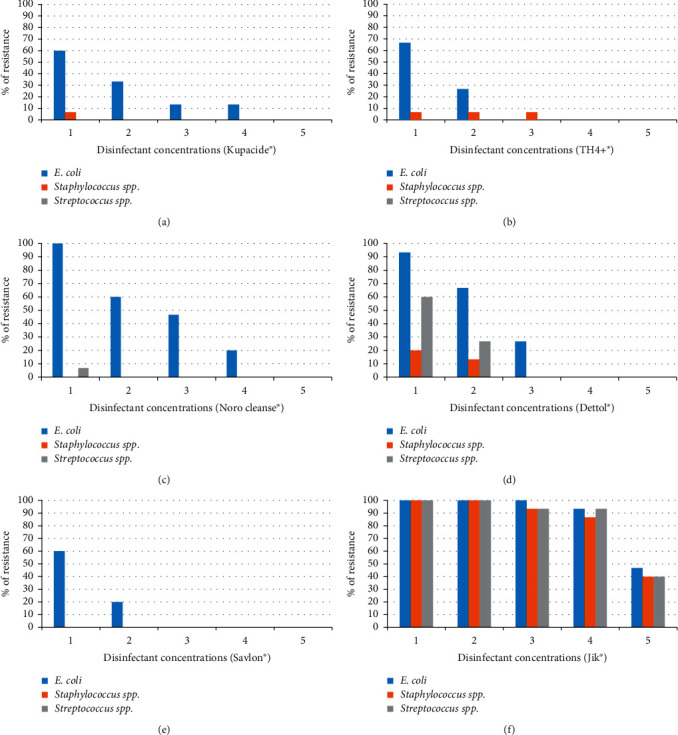
Resistance of isolates to the tested disinfectants.

*Escherichia coli* isolates showed a resistance rate of 60% at the lowest concentration used (concentration x1/4); 33.3% at concentration x1/2; 13.3% at recommended concentration; 13.3% at concentration x2; and 0% at concentration x4. *Staphylococcus* isolates were all susceptible at concentration x1/2, and recommended concentration, x2 and x4; they showed 6.7% resistance at concentration x1/4. All the *Streptococcus* isolates were susceptible to Kupacide^®^ at all concentrations. For TH4+^®^, *Escherichia coli* isolates were resistant at 66.7% and 26.7% at concentration x1/4 and x1/2, respectively; all were susceptible at recommended concentration, concentrations x2 and x4. Fourteen (93.3%) of the tested *Staphylococcus* isolates were susceptible to all concentrations; only one isolate (6.7%) was resistant to concentration x1/4 and x1/2 and recommended concentration. All tested *Streptococcus* isolates were susceptible to all concentrations. Figures 1(a) and 1(b) show the resistance pattern of the isolates for Kupacide^®^ and TH4+^®^, respectively.

As shown by Figure 1(c), *Escherichia coli* isolates showed resistance of 100% to Noro cleanse^®^ at concentration x1/4; 60% at concentration x1/2; 46.7% at recommended concentration; 20% at concentration x2, and 0% at concentration x4. All tested *Staphylococcus* isolates were susceptible to Noro cleanse^®^ at all concentrations. *Streptococcus* isolates were susceptible at all concentrations except for concentration x1/4, where they showed resistance of 6.7%.

At lowest concentration x1/4 of Dettol^®^, *E. coli* isolates were resistant at 93.3%, *Staphylococcus* isolates were resistant at 20%, and *Streptococcus* isolates were resistant at 60%; at concentration x1/2, *E. coli* isolates were resistant at 66.7%: *Staphylococcus* isolates at 13.3% and *Streptococcus* isolates at 26.7%. At the manufacturer's recommended concentration, *E. coli* isolates were resistant at 26.7%; *Staphylococcus* and *Streptococcus* isolates were all susceptible. At higher concentrations (x2 and x4), all the isolates were susceptible to Dettol^®^. Figure 1(d) shows resistance of isolates to Dettol^®^.

All the tested *Staphylococcus* and *Streptococcus* isolates were susceptible to Savlon^®^ at all concentrations; *E. coli* showed resistance of 60% at lowest concentration (x1/4) and 20% at concentration x1/2.

**Figure 2:**
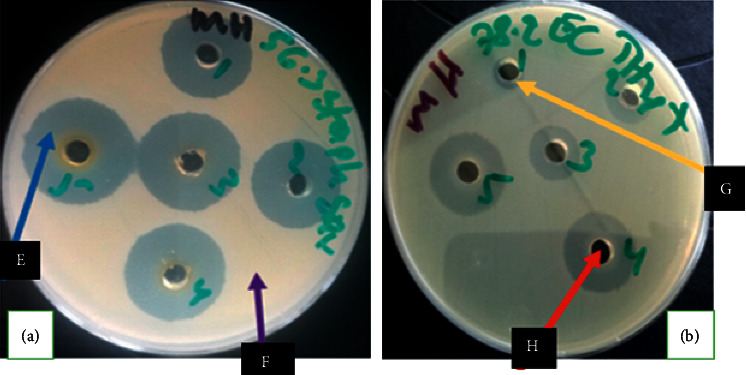
Photograph showing agar diffusion disinfectant susceptibility results. Inhibition zones are pointed by blue arrow (E); wells which were containing the disinfectant are pointed by the red arrow (H). Purple arrow (F) is showing confluent bacterial (of *Staphylococcus* isolate) growth, and yellow arrow (G) is showing resistance (no inhibition zone).

Isolates showed higher resistance to Jik^®^ compared to other disinfectants tested. *E. coli*, *Staphylococcus*, and *Streptococcus* were resistant at 100% at concentration x1/4 and x1/2. At the manufacturer's recommended concentration, all *E. coli* isolates were resistant (100%); *Staphylococcus* and *Streptococcus* isolates were both resistant at 93.3%. At concentration x2, *E. coli*, *Staphylococcus*, and *Streptococcus* were resistant at 93.3%, 86.7%, and 93.3%, respectively; at the highest concentration used x4, *E. coli* isolates were resistant at 46.7%; *Staphylococcus* isolates at 40% and *Streptococcus* isolates at 40%. Figures 1(e) and 1(f) show resistance patterns of the test isolates to Savlon^®^ and Jik^®^, respectively, with respect to various concentrations.  Figure 2 shows agar well diffusion susceptibility/resistance results of some disinfectants against the isolates. The percentage of resistance for the tested isolates at recommended user concentration is given in Table 2.

*E. coli* reference strain, ATCC 259222, was susceptible at all concentrations for Savlon^®^ and Noro cleanse^®^. It was resistant at x1/4 to Dettol^®^ and susceptible to the remaining concentrations (x1/2, recommended concentration x2 and x4); the strain was resistance to Kupacide^®^ at x1/4 and x1/2 and susceptible at remaining concentrations (recommended concentration, x2 and x4). It was resistant to Jik^®^ and TH4+^®^ at x1/4 and x1/2 and recommended concentration but susceptible at x2 and x4. The reference strain *Staphylococcus* ATCC 25922 was susceptible to all concentrations to disinfectants Kupacide^®^, TH4+^®^, Noro cleanse^®^, Dettol^®^, and Savlon^®^; however, it was resistant to Jik^®^ at x1/4, x1/2, recommended concentration and x2 but susceptible to concentration x4.

Some isolates expressed resistance to more than one disinfectant at recommended concentration. 11 (73.3%) of *Escherichia coli* isolates showed resistance to more than one disinfectant, and 2 (13.3%) were even resistant to 4 disinfectants. Only one (6.7%) *Staphylococcus* isolate showed resistance to more than one disinfectant. No *Streptococcus* isolate showed resistant to more than one disinfectant.  Table 3 shows the disinfectant combinations the respective bacteria were multiresistant to.

## 4. Discussion

In this study, six different disinfectants which are currently used in poultry production, other food-producing units, and humans (homes and health facilities) were tested against three isolated bacteria which were *E. coli*, *Staphylococcus*, and *Streptococcus*. The isolates showed high resistance level/percentage to Jik^®^ not only at and below the recommended user concentration but also at the two higher concentrations (x2 and x4). This could have been anticipated by the fact that Jik^®^ is frequently used as a disinfectant or as the result of its chemical composition (3.85% m/v of sodium hypochlorite). Sodium hypochlorite is a halogen that acts by denaturing bacterial proteins; for a disinfectant to work properly, it must cross the outer membrane of the bacteria and reach the target site [6]. This may be one of the reasons why Jik^®^ was less effective since it may not have activities to disrupt cell wall or membrane that can help its easy absorption into bacterial cells; hence, it was less effective in killing the bacteria at low concentrations. In the study conducted by Wanja et al. [13], the tested bacteria isolate also showed resistance to sodium hypochlorite at recommended user concentration.

In their study, Njagi et al. [2] showed that all the tested isolates, which included *E. coli*, were resistant (100%) to disinfectant C (which was containing sodium hypochlorite) at recommended user concentration of 2.5% of sodium hypochlorite though this concentration was a bit higher compared to the one recommended in this study (2.27%). It may, therefore, be advisable to use sodium hypochlorite in combination with another disinfectant or use it at higher concentration than recommended so that it can give better results.

46.7% of *E. coli* isolates were resistant to Noro cleanse^®^ at recommended user concentration and 20% at x2 which is the double the recommended concentration. However, *Staphylococcus* and *Streptococcus* isolates were 100% susceptible at recommended user concentration and also at x1/2, which was obtained by double diluting the recommended user concentration. Noro cleanse^®^ being composed by coco-benzyl-dimethyl-ammonium chloride that denatures proteins and interferes with membrane integrity; however, it is known to be ineffective against Gram-negative bacteria, when used alone [6], in combination with glutaraldehyde which cause protein crosslinking [6], the effect of these chemicals against the tested bacteria was increased at recommended used concentration, despite that, *E. coli* was less responsive, and this may be due to its cell membrane which was not permissive for the entrance of disinfectant.

In the study done by Njagi et al. [2], similar disinfectants to the ones used in current study were tested: they used glutaraldehyde which is similar in composition to Noro cleanse^®^ in this study and chloroxylenol which is similar to Dettol^®^. However, the results obtained back then are quite different from the current findings where isolates as being 100% resistant at recommended user concentration with 25% resistance at higher concentration for both Dettol^®^ and Noro cleanse^®^. *E. coli* was resistant at 46.7% to Noro cleanse^®^ and 26.7% resistance to Dettol^®^. Dettol^®^ is composed of chloroxylenol 4.8%, one of the groups of halophenol, which works by denaturing proteins, altering cell wall permeability and causing cell leakage [6]. It is known as good disinfectant for both Gram-positive and Gram-negative bacteria with greater effect on Gram-positive ones [14]; hence, it is not surprising that *Staphylococcus* and *Streptococcus* isolates were 100% susceptible to Dettol^®^ at recommended user concentration and only 26.7% of *E. coli* were resistant.

Savlon^®^ has shown high activity against the isolates compared to other disinfectants, which manifested its strong activity against both the Gram-negative and Gram-positive bacteria. Its effectiveness can be due to its formulation which is chlorhexidine gluconate 0.3 gram (a biguanide that alters membrane permeability and damage to the outer cell layers of bacteria); cetrimide 3.0 gram (an ammonium quaternary compound, which works by denaturing proteins and interfering with bacterial cell membrane integrity [6]); and N-propylalcohol 2.84% m/v, an alcohol which is known to cause membrane damage, protein denaturation, interference with metabolism, and resultant cell lysis [15]. The combination of all these components which have different effects on bacteria may be the reason why this disinfectant showed larger inhibition zones than others, an indication of high antibacterial activity. The study done by Stringfellow et al. [7] has shown the effectiveness of chlorhexidine against bacteria, which is not different from this case of Savlon^®^.

With respect to Kupacide^®^, *Streptococcus* isolates were 100% susceptible at all concentrations; *Staphylococcus* isolates were susceptible at 100% at all concentrations except at x1/4, while *E. coli* isolates were resistant at 13.3%. With respect to TH4+^®^, at recommended user concentration, *Staphylococcus* isolates were resistant at 6.7%, while *E. coli* isolates were susceptible. The active ingredients of the two disinfectants may have been among the factors which influenced this outcome. Both TH4+^®:^ and Kupacide^®^ are composed of quaternary ammonium compounds (QACs) and glutaraldehyde; as mentioned before, QACs work mainly by disrupting the membrane integrity and glutaraldehyde causes crosslinking of proteins; hence, their mode of action is almost the same. However, it has been shown that some Gram-negative bacteria can adapt to benzalkonium chloride (found in Kupacide^®^) and thus become resistant to it [16]; this may also have contributed to the 13.3% resistance rate in *E. coli*, at recommended user concentration as well as at double that concentration (x2).

Apart from chemical composition and concentration, different factors are known that cause resistance of bacteria to a particular disinfectant; they include time of exposure, presence of interfering compounds of organic and inorganic matter, temperature, and type of targeted microorganisms (presence of biofilm or inoculum of the organism) and their concentration (inoculum) among others [17]. In this study, all the tested disinfectants were subjected to the same temperature (37°C), and the test was conducted on Muller-Hinton agar media which were not containing other compounds and incubated overnight. Thus, the mentioned factors had minimal, if any, interference on the tests.

As found in this study, there is a difference in mode of reaction of Gram-negative, *E. coli*, and Gram-positive, S*taphylococcus* and *Streptococcus*, isolates, to disinfectants; *E coli* isolates were more resistant to the disinfectants compared to *Staphylococcus* and *Streptococcus* isolates. Investigations have shown that Gram-negative bacteria tend to be less responsive to disinfectant compared to Gram-positive ones; however, Gram positives that form spores and few exceptions such as mycobacteria are less responsive [9]. This is mainly due to intrinsic factors and difference in cell structure between Gram negatives and Gram positives, and it has been shown that the cell wall of Gram-positive bacteria does not act as barrier for disinfectants unlike the for Gram-negative ones [6].

Resistance to disinfectants can also be genetically encoded by the organism [18, 19] or be carried by plasmids [18, 20]. So, there is a possibility that some of the resistance recorded in this study may be due to presence of resistance genes for the particular disinfectant. There is a concern that use of nonantibiotic antimicrobial agents could induce biocide resistance and lead to selection of antibiotic resistant bacteria especially when used at lower concentration which is unable to kill them [9]. In the study done by Igizeneza [21], the same bacteria used in this study was also subjected to different antibiotics and showed resistance to them.

The findings from this study indicated that (1) the higher the concentration of disinfectant, the more effective it works and (2) active ingredients of disinfectants play a major role in killing and inhibiting bacterial growth. In general, Gram-positive bacteria were found to be more susceptible to disinfectants than Gram-negative bacteria; disinfectants which controlled Gram-negative bacteria were those that had active ingredients which act on the cell wall. Disinfectants which contain different varieties of active ingredients that target different sites of bacterial cells were found to be more effective in controlling both Gram-negative and Gram-positive bacteria. Thus, selection of effective disinfectants is important; this emphasizes the importance of carrying out disinfectant susceptibility testing before use. It is recommended that the responsible organization, the Clinical and Laboratory Standards Institute, should provide a range of cutoff points for different disinfectants which are commonly used so that people can be able to compare the effective results. Results of this study will contribute towards data on extent of disinfectant resistance in the country. It will also inform policy makers and help guide the setting of control guidelines towards responsible usage of disinfectants.

## Figures and Tables

**Table 1:** Respective active ingredients of the tested disinfectants.

Disinfectants	Active ingredients of the disinfectant
Kupacide^®^	Glutaraldehyde 15% v/v; benzalkonium chloride 10% v/v
Th4+^®^	Didecyl dimethyl ammonium HCl 18.75 gram; dioctyl dimethyl ammonium HCl 18.75 g; octyl decyldimethyl ammonium HCl 37.5 gram; alkyl dimethyl ammonium HCl 50 gram; and glutaraldehyde 62.50 gram
Noro cleanse^®^	Glutaraldehyde 15% w/v; coco-benzyl-dimethyl-ammonium chloride (QAC) 10% w/v
Dettol^®^	Chloroxylenol 4.8%
Savlon^®^	Chlorhexidine gluconate 0.3 gram; cetrimide 3.0 gram; N-propylalcohol 2.84% m/v
Jik^®^	3.85% m/v of sodium hypochlorite

**Table 2:** Percent resistance of the tested isolates at recommended user concentration, for *n* = 5.

		Disinfectants											
		Jik^®^		Dettol^®^		Savlon^®^		Kupacide^®^		TH4+^®^		Noro cleanse^®^	
Isolates	Slaughterhouses	*n*	R (%)	*n*	R (%)	*n*	R (%)	*n*	R (%)	*n*	R (%)	*n*	R (%)
*E. coli*	Kariokor	5	100	1	20	0	0	2	40	0	0	5	100
	Burma	5	100	2	60	0	0	3	60	0	0	2	40
	Kangemi	5	100	1	20	0	0	1	20	0	0	0	0

*Staphylococcus* isolates	Kariokor	5	100	0	0	0	0	0	0	1	20	0	0
	Burma	5	100	0	0	0	0	0	0	0	0	0	0
	Kangemi	4	80	0	0	0	0	0	0	0	0	0	0

*Streptococcus* isolates	Kariokor	4	80	0	0	0	0	0	0	0	0	0	0
	Burma	5	100	0	0	0	0	0	0	0	0	0	0
	Kangemi	5	100	0	0	0	0	0	0	0	0	0	0

**Table 3:** Multiple resistance to disinfectants at recommended user concentration.

Bacteria	Disinfectants at recommended concentration	Frequency of appearing %
*E. coli*	Jik^®^, Kupacide^®^, and Noro cleanse^®^	2/15 (13.3%)
	Jik^®^, Dettol^®^, Kupacide^®^, and Noro cleanse^®^	2/15 (13.3%)
	Jik^®^ and Noro cleanse^®^	3/15 (20%)
	Jik^®^ and Kupacide^®^	2/15 (13.3%)
	Jik^®^ and Dettol^®^	2/15 (13.3%)

*Staphylococcus* spp.	Jik^®^ and TH4+^®^	1/15 (26.7%)

## Data Availability

The authors would like that the findings from this investigation can reach other researchers as well as the entire community. Also, we hope that our recommendation will reach the responsible institutions so that they can be taken into consideration.
